# Quantitative Spermidine Detection in Cosmetics using an Organic Transistor‐Based Chemical Sensor

**DOI:** 10.1002/open.202400098

**Published:** 2024-09-05

**Authors:** Yui Sasaki, Kohei Ohshiro, Miyuki Kato, Hikaru Tanaka, Akari Yamagami, Kazutake Hagiya, Tsuyoshi Minami

**Affiliations:** ^1^ Institute of Industrial Science The University of Tokyo 4-6-1, Komaba Meguro-ku 153-8505 Tokyo Japan; ^2^ JST, PRESTO 4-1-8 Honcho Kawaguchi 332-0012 Saitama Japan; ^3^ Corporate Research Center, Toyobo Co., Ltd. 2-1-1 Katata Otsu 520-0292 Shiga Japan

**Keywords:** Sensors, Amines, Organic transistor, Cosmetic analysis

## Abstract

Spermidine is an essential biomarker related to antiaging. Although the detection of spermidine levels is in high demand in life science fields, easy‐to‐use analytical tools without sample purification have not yet been fully established. Herein, we propose an organic field‐effect transistor‐based chemical sensor for quantifying the spermidine concentration in commercial cosmetics. An extended‐gate structure was employed for organic field‐effect transistor (OFET)‐based chemical sensing in aqueous media. A coordination‐bond‐based sensing system was introduced into the OFET device to visualize the spermidine detection information through changes in the transistor characteristics. The extended‐gate‐type OFET has shown quantitative responses to spermidine, which indicates sufficient detectability (*i. e*., the limit of detection for spermidine: 2.3 μM) considering actual concentrations in cosmetics. The applicability of the OFET‐based chemical sensor for cosmetic analysis was validated by instrumental analysis using high‐performance liquid chromatography. The estimated recovery rates for spermidine in cosmetic ingredient products (108–111 %) suggest the feasibility of cosmetic analysis based on the OFET‐based chemical sensor.

## Graphical abstract

An extended‐gate‐type organic transistor can be used to quantify spermidine levels in cosmetic ingredient products without complicated sample purification. The accurate detection results, validated by HPLC, indicate the potential of organic transistors as platforms for cosmetic analysis.

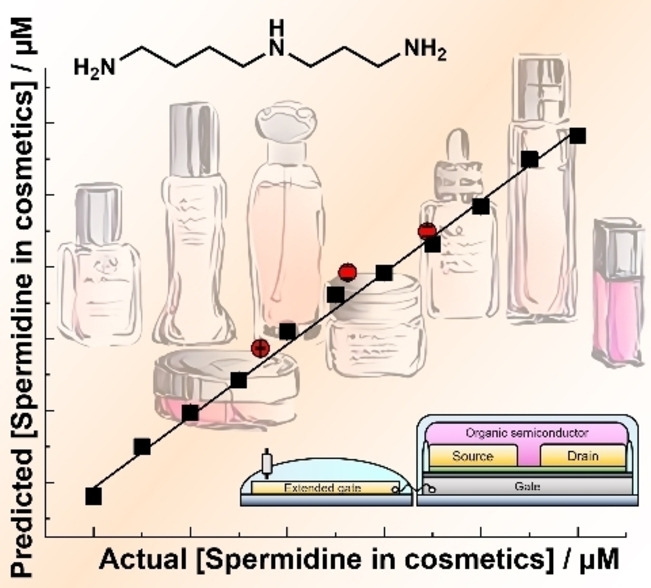

## Introduction

Polyamines are essential biological species involved in cell growth, gene expression, protein synthesis, and autophagy,^[1]^ the physiological functions of which are closely associated with biological aging (or senescence).^[2]^ Therefore, polyamines are crucial markers for examining aging‐associated pathologies (*e. g*., cardiovascular disease, cancer, and metabolic disorders).^[3]^ In particular, spermidine modulates aging and suppresses age‐related diseases.^[4]^ In this regard, the association between spermidine and biological aging has received attention in life science fields from a health and beauty perspective.^[5]^ Although spermidine assessment is in high demand in various research fields, easy‐to‐use methods for monitoring spermidine levels have not been satisfactorily established. Motivated by this demand, we propose a chemical sensor device based on an organic field‐effect transistor (OFET) to quantify the spermidine concentration in commercial cosmetic ingredient products.

OFETs are electronic devices with gate, source, and drain electrodes; a dielectric; and an organic semiconductive layer, which shows unique switching properties upon the application of voltages.^[6]^ Solution processability, by employing highly soluble materials for device fabrication, can accelerate the development of OFET‐based chemical sensors using printing technologies.^[7]^ The transistor characteristics (*i. e*., drain currents and threshold voltages) are manipulated by the gate potentials, indicating that a gate electrode can be used as a sensing unit by functionalizing it with recognition materials.^[8]^ An extended‐gate structure, which isolates the sensing unit from the operation unit (*i. e*., an OFET), allows the stable electrical detection of analytes in aqueous media.^[8]^ Extended‐gate gold (Au) electrodes can easily be functionalized with molecular recognition materials such as self‐assembled monolayers (SAMs).^[9]^ The transistor characteristics depend on the surface potential of the extended‐gate electrode functionalized with the SAM‐based molecular recognition materials upon analyte detection.^[10]^ Recently, we proposed a new strategy for polyamine detection based on coordination bonds using an extended‐gate‐type OFET.^[11]^ However, the applicability of this sensing method has not been fully evaluated in real‐sample analyses. In this study, an OFET‐based chemical sensor for spermidine detection was used for cosmetic analysis without sample purification.

**Figure 1:**
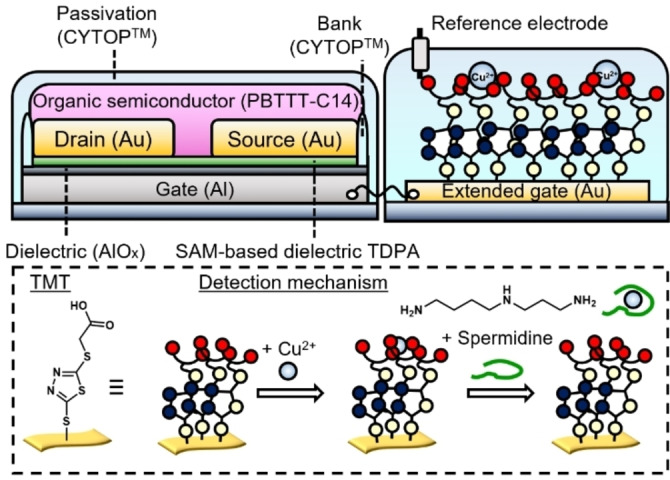
Schematic illustration of the extended‐gate‐type OFET chemical sensor for spermidine. The extended‐gate Au electrode was functionalized with TMT to accumulate Cu^2+^ ions. Spermidine detection is performed by a competitive assay among TMT, the Cu^2+^ ions, and the target spermidine.

Copper(II) ions (Cu^2+^) were selected as the charge manipulators to create a coordination‐bond‐based sensing system using an OFET (*vide infra*).[^11^, ^12^] 2‐Carboxymethylthio‐5‐mercapto‐1,3,4‐thiadiazole (TMT) functionalized on an extended‐gate electrode can coordinate with Cu^2+^ ions.[^11^, ^13^] However, the binding affinity between TMT and Cu^2+^ ions is lower than that between Cu^2+^ ions and spermidine. Therefore, the Cu^2+^ ions accumulated on the TMT‐attached extended‐gate electrode caused changes in the transistor characteristics with the addition of spermidine because of the removal of Cu^2+^ ions from the TMT‐Cu^2+^ complex (Figure 1). In addition, the electron density of the heteroaromatic structure of TMT was higher than that of the mercapto‐benzoic acid derivatives.[^14^, ^15^] Therefore, the proposed sensing system can induce distinct surface potential changes in the extended‐gate electrode by detecting spermidine in cosmetic ingredient products.

## Experimental

### Fabrication and Operation of the OFET‐Based Chemical Sensor

The designed OFET device consisted of an aluminum (Al) gate, Au source, and drain electrodes. A double dielectric layer composed of aluminum oxide (AlOx) and tetradecylphosphonic acid (TDPA)^[16]^ was employed to operate the OFET at low voltages (< |3| V) for analyte detection in aqueous media. The semiconducting layer was fabricated via a drop‐casting approach using a solution‐processable polymer semiconductor (*i. e*., poly{2,5‐bis(3‐tetradecylthiophen‐2‐yl)thieno[3,2‐*b*]thiophene}, PBTTT−C14).^[17]^ The surface of the OFET device was fully covered with an amorphous fluorinated polymer material (*i. e*., CYTOP^TM^) to passivate the semiconducting layer. The details of the device fabrication process are summarized in the Supporting Information (SI) (Figure S1).

The extended‐gate electrode was functionalized stepwise with TMT and Cu^2+^ ions. An extended‐gate Au electrode (15 mm^2^ area, 100 nm thickness) was formed on a polyethylene naphthalate film by vacuum thermal deposition (SVC‐700TMSGS, Sanyu Electron Co., Ltd.). The Au electrode was gently rinsed with Milli‐Q water and ethanol and then dried under a N_2_ gas flow. The Au electrode was further treated using a UV‐O_3_ cleaner (ASUMI‐GIKEN, ASM401 OZ) for 10 min before subsequent modification. The extended‐gate Au electrode was immersed in a methanol solution of TMT (10 mM) for 1 h at 25 °C under ambient conditions. The Au electrode surface was gently rinsed with methanol and dried under a N_2_ gas flow. Next, the Au electrode functionalized with TMT was further immersed into a buffer solution made of 4‐(2‐hydroxyethyl)‐1‐piperazineethanesulfonic acid (100 mM, pH 7.4 at 25 °C) containing Cu(ClO_4_)_2_ (1 mM) for 1 h at 25 °C under ambient conditions. After this period, the electrode was rinsed with ultrapure water and dried under a N_2_ gas flow. The extended‐gate electrode was connected to an OFET using a conductive cable for chemical sensing. A reference electrode (Ag/AgCl, model RE‐1B, BAS, Inc.) was used to apply the gate voltage (Figure 1).

The basic characteristics of the fabricated OFET were evaluated using a source meter (2612 B, Keythley) under ambient conditions. The transfer characteristics were obtained by scanning from 0.5 to −3 V as the gate voltage (*V*_GS_) and −2 V as the drain‐source voltage (*V*_DS_). The output characteristics were recorded at 0 to −3 V (step: −1 V) as *V*_GS_ and 0 to −3 V as *V*_DS_. The OFET exhibited switching properties under ambient conditions (Figure S2).

### High‐Performance Liquid Chromatography Analysis

The concentrations of representative polyamines (*i. e*., putrescine, spermidine, and spermine) were validated using high‐performance liquid chromatography (HPLC) (Figure S3). Standard stock solutions of polyamines were prepared using HCl_aq._ (0.1 M). The concentrations of each stock solution were 99.8 mM for putrescine, 124.1 mM for spermidine, and 53.9 mM for spermine, respectively. A standard mixture solution was prepared by using the stock solutions (putrescine (100 μL, 99.8 mM), spermidine (80 μL, 124.1 mM), spermine (185 μL, 53.9 mM)), and HCl_aq._ (635 μL, 0.1 M). The concentration of each polyamine in the standard mixture was 10 mM. The standard mixture was diluted with HCl_aq._ (0.1 M) to adjust the concentrations of each polyamine to 0.3, 1, 3, 10, 30, and 100 μM.

The target polyamines were labeled using dansyl derivatization. Cosmetic ingredients, including polyamines (PHYTOPOLYAMINE^TM^‐S (solution) and PHYTOPOLYAMINE^TM^‐SP (powder)), were dissolved in HCl_aq_ (0.1 M). The concentrations of the aqueous solutions of PHYTOPOLYAMINE^TM^‐S and PHYTOPOLYAMINE^TM^‐SP were adjusted to 200 and 10 mg/mL, respectively. A reference sample made of 1,7‐diaminoheptane (50 μL, 10 μM), dansyl chloride in acetone (300 μL, 1 wt%), and a saturated aqueous solution of sodium carbonate (50 μL) was injected into the polyamine mixture solution (50 μL). The obtained solution was incubated at 70 °C for 15 min. After this period, the proline solution (50 μL, 10 wt%) was added to the mixture and incubated at 70 °C for 5 min. When a yellow solution was obtained, toluene was added, followed by vortexing for 30 s. Centrifugation was performed at 15000 rpm for 10 min at 15 °C (HIGH SPEED MICRO CENTRIFUGE MX‐150, TOMY), and a toluene layer was removed. The obtained solution was dried at 40 °C for 1 h under N_2_ gas flow using an aluminum block dry bath (Dry ThermoUnit DTU‐1BN, TAITEC). Subsequently, an aqueous acetonitrile solution (1.0 mL, acetonitrile/water=1 : 1 vol./vol.) was added to the dried residue. The mixture was centrifuged at 15000 rpm for 2 min at 15 °C. The supernatant was subjected to HPLC analysis.

The employed HPLC apparatus (Shimadzu) was equipped with a CBM‐20 A communications bus module, a Shimpack GIST C18 column (4.6 mm×250 mm, 3 μm), Shimpack GIST C18 guard column (4.6 mm×50 mm, 3 μm), a DGU‐20 A3 degasser, an LC‐20AD pump, a SIL‐10ADvp autosampler, a CTO‐20 A column oven, and an RF‐10AXL fluorescence detector. HPLC measurement conditions were as follows: flowrate, 0.8 mL/min; excitation and emission wavelengths of the fluorescence detector, 340 and 515 nm; column oven temperature, 40 °C; and injection volume, 5 μL. The mobile phase consisted of acetonitrile and Milli‐Q water with gradient flow. The acetonitrile concentration was increased from 65 vol.% to 100 vol.% within 15 min and then kept at 100 vol.% until it was reduced to 65 vol.% at 20 min and ended at 30 min. Chromatographic data were acquired and processed using Shimadzu LabSolutions software.

## Results and Discussion

### Establishment of a Calibration Line using the OFET‐Based Chemical Sensor for Spermidine Levels

**Figure 2:**
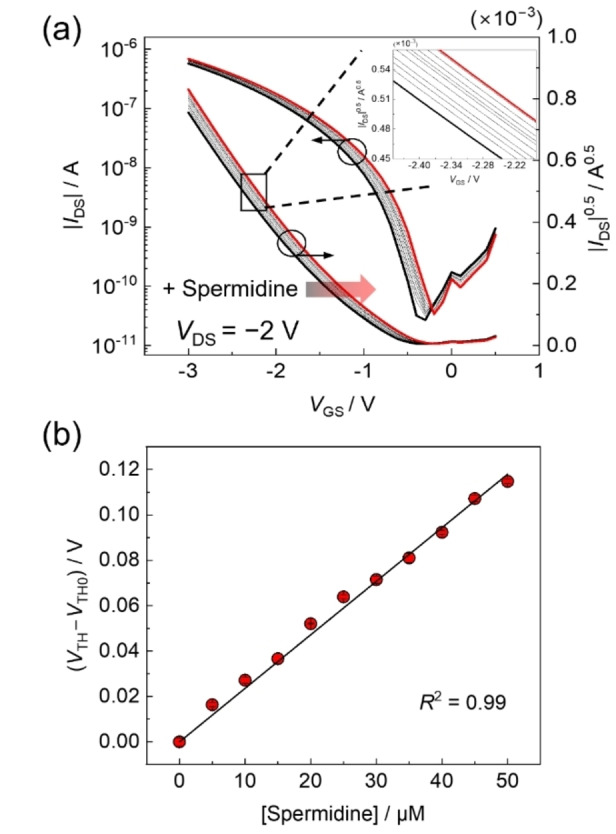
(a) Transfer curves of the OFET‐based chemical sensor upon the addition of spermidine in a CHES buffer solution (50 mM) containing NaCl (10 mM) at pH 8.5. Transistor characteristics at each spermidine concentration were recorded after incubation for 5 min at 25 °C. (b) The correlation between the spermidine concentration and threshold voltage (*V*_TH_). The terms *V*_TH0_ and *V*_TH_ indicate threshold voltages before and after the addition of spermidine at different concentrations. The correlation was obtained by collecting *V*_TH_s at *V*_GS_=−2 V.

A calibration line for spermidine analysis in cosmetics was established using transistor characteristics upon the addition of spermidine at different concentrations in a buffer solution containing *N*‐cyclohexyl‐2‐aminoethanesulfonic acid (CHES, 50 mM) and NaCl (10 mM) at pH 8.5. The basic conditions were adjusted based on the superior coordination of spermidine and Cu^2+^ ions.^[18]^ Figure 2(a) shows the changes in the transistor characteristics upon the addition of spermidine. According to the detection principle based on a competitive assay, Cu^2+^ ions were removed from the Cu^2+^‐TMT complex by coordination with the target spermidine. The removal of cationic species from the extended‐gate electrode caused changes in the surface potential, resulting in positive shifts in the transfer characteristics. The correlation between the spermidine concentration and threshold voltage (*V*_TH_) in Figure 2(b) suggests that the shift in transistor characteristics quantitatively corresponds to the spermidine concentration. The detection limit for spermidine (2.3 μM) was determined by the 3σ method.^[19]^ Considering the actual concentrations of spermidine in cosmetics, the sensitivity of the OFET‐based chemical sensor was sufficient for cosmetic analysis.

### Determination of Spermidine Levels in Cosmetic Ingredient Products

**Figure 3:**
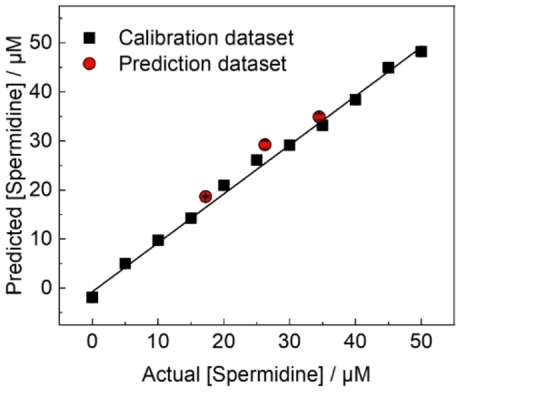
Spike and recovery test for spermidine contained in a commercial cosmetic ingredient product (*i. e*., PHYTOPOLYAMINE^TM^‐S).

We performed a real‐sample analysis and established a calibration line using different extended‐gate electrodes. The real‐sample analysis without any purification was performed for the main target spermidine using the extended‐gate‐type OFET. Two types of cosmetic ingredient products (*i. e*., PHYTOPOLYAMINE^TM^‐S (solution) and PHYTOPOLYAMINE^TM^‐SP (powder)) were used as real samples to evaluate the applicability of the OFET‐based sensing method. For validation, spermidine concentrations in commercial cosmetic ingredient products were estimated using HPLC analysis (Figure S4 and Table S1). The concentrations of putrescine and spermine were lower than those of spermidine, implying that the effect of these amines in response to spermidine was weak. The datasets (red circles) for spermidine in PHYTOPOLYAMINE^TM^‐S were closely distributed on the established calibration line (Figure 3). The recovery rates were estimated to be 108–111 % (Table 1). Moreover, an OFET‐based chemical sensor was used to quantify spermidine levels in PHYTOPOLYAMINE^TM^‐SP. The determined recovery rates were 105–116 % (Table S2). This study suggests the potential of the OFET‐based chemical sensor for the evaluation of spermidine levels in cosmetic ingredient products with high accuracy.

**Table 1:** Spike and recovery test results for spermidine contained in a commercial cosmetic ingredient (*i. e*., PHYTOPOLYAMINE^TM^‐S).

Actual [Spermidine] (μM)	Found [Spermidine] (μM)	Recovery rate (%)
17.23	18.7±0.2	108
26.24	29.2±0.8	111
34.46	38.6±0.8	110

## Conclusions

Spermidine is a representative ingredient contained in cosmetics because of its antiaging effects. Although the interest in spermidine evaluation has increased in life science fields, an easy‐to‐use analytical method for spermidine detection is challenging. Therefore, we designed a chemical sensor device based on an OFET for cosmetic analysis without complicated sample purification. A competitive detection system based on coordination bonds was introduced into an extended‐gate‐type OFET to visualize spermidine detection information through changes in transistor characteristics. The fabricated OFET‐based chemical sensor showed quantitative detection of the target spermidine with a low limit of detection value (2.3 μM). The sensitivity of the OFET‐based sensor for spermidine was sufficient considering its concentrations in cosmetic ingredient products. The applicability of the OFET‐based chemical sensor was revealed by the spike and recovery rates of spermidine in commercial cosmetics. The accuracy of the OFET‐based chemical sensor was validated using conventional HPLC. In addition, because OFETs can be fabricated on plastic films using printing technologies,^[7d–f]^ flexible chemical sensors may allow the monitoring of spermidine levels in the skin.

## Conflict of Interests

The authors declare no conflict of interest.

1

## Supporting information

As a service to our authors and readers, this journal provides supporting information supplied by the authors. Such materials are peer reviewed and may be re‐organized for online delivery, but are not copy‐edited or typeset. Technical support issues arising from supporting information (other than missing files) should be addressed to the authors.

Supporting Information

## Data Availability

The data that support the findings of this study are available in the supplementary material of this article.
