# Supplementary material for: Quantitative Spermidine Detection in Cosmetics using an Organic Transistor‐Based Chemical Sensor
Source: ChemistryOpen. 2024 Sep 5;13(10):e202400098. doi: 10.1002/open.202400098 (PMC12056924; doi:10.1002/open.202400098)
Supplement: Supplementary file 1 — Supporting Information [file OPEN-13-e202400098-s001.pdf]

ChemistryOpen

Supporting Information

Quantitative Spermidine Detection in Cosmetics using an Organic Transistor-Based Chemical Sensor

Yui Sasaki, Kohei Ohshiro, Miyuki Kato, Hikaru Tanaka, Akari Yamagami, Kazutake Hagiya, and Tsuyoshi Minami*

Supporting Information

Quantitative Spermidine Detection in Cosmetics using an Organic Transistor-based Chemical Sensor

Yui Sasaki,[a, b] Kohei Ohshiro,[a] Miyuki Kato,[a] Hikaru Tanaka,[c] Akari Yamagami,[c] Kazutake Hagiya,[c] and Tsuyoshi Minami*[a]

[a] Institute of Industrial Science, The University of Tokyo, 4-6-1 Komaba, Meguro-ku, Tokyo 153-8505, Japan. E-mail: tminami@g.ecc.u-tokyo.ac.jp

[b] JST, PRESTO, 4-1-8 Honcho, Kawaguchi, 332-0012 Saitama, Japan.

[c] Corporate Research Center, Toyobo Co., Ltd., 2-1-1 Katata, Otsu, 520-0292 Shiga, Japan.

Contents

1. Reagents and materials **S2**

2. Fabrication and evaluation of an extended-gate type OFET sensor **S2**

3. HPLC analysis **S4**

4. Real-sample analysis using the OFET-based chemical sensor **S5**

Reference **S5**

1. Reagents and materials

Reagents and solvents obtained from commercial suppliers were applied without further purification. The reagents purchased from Merck KGaA were copper(II) perchlorate hexahydrate ($Cu(ClO_4)_2 \cdot 6H_2O$), spermidine, and spermine tetrahydrochloride. 1,4-Diaminobutane dihydrochloride (putrescine), 1,7-diaminoheptane, dansyl chloride, L-proline, and tetradecylphosphonic acid (TDPA) were obtained from Tokyo Chemical Industry Co. Ltd. Sodium chloride, hydrochloric acid (HCl), ethanol, toluene, acetone, 2-propanol, and methanol were purchased from Kanto Chemical Co. Inc. Sodium carbonate and poly{2,5-bis(3-tetradecylthiophen-2-yl)thieno[3,2-*b*]thiophene} were obtained from FUJIFILM Wako Pure Chemical Industries, Ltd. and Merck KGaA, respectively. Materials supplied by AGC Co. Ltd. were an amorphous fluorinated polymer CYTOP™ (model: CTX-809M) and perfluorotributylamine. Glass substrates (model: Eagle XG, 2 cm × 2.5 cm) obtained from Corning, Inc. was used for device fabrication. Materials for vacuum deposition were aluminum (Al) wire (1ϕ) and gold (Au) particle, which were purchased from Furuuchi Chemical Co., Ltd and Tanaka Kikinzoku Kogyo Co., Ltd, respectively. *N*-Cyclohexyl-2-aminoethanesulfonic acid (CHES) and 4-(2-hydroxyethyl)-1-piperazineethanesulfonic acid (HEPES) were purchased from Dojindo Laboratories. 2-Carboxymethylthio-5-mercapto-1,3,4-thiadiazole (TMT), PHYTOPOLYAMINE™-S, PHYTOPOLYAMINE™-SP, and polyethylene naphthalate (PEN) films were supplied by Toyobo Co., Ltd. Aqueous solutions for all experiments were prepared using a Milli-Q water system (18.2 MΩ・cm).

2. Fabrication and evaluation of an extended-gate type OFET sensor

2.1 OFET fabrication

The eagle glass substrate (2 cm × 2.5 cm) was treated with piranha solution (H_2SO_4:H_2O_2 = 4:1 (v/v)) before device fabrication. A gate electrode made of Al (30 nm in thickness) was fabricated using a vacuum thermal deposition apparatus (SVC-700TMSGS, Sanyu Electron Co., Ltd.). Shadow masks were used for patterning. The surface of Al electrodes was activated by a reactive ion etching (RIE) process (SAMCO RIE-10NR) to obtain an aluminum oxide (AlOx) dielectric layer. Subsequently, the glass substrate was entirely covered with a fluorinated polymer solution (CLT-809M in CT-Solv.180, ratio 1:1 (v/v)) using a spin-coater apparatus (MIKASA SPINCOATER 1H-D7). The glass substrate was baked at 110 °C for 10 min in the inert atmosphere glovebox (UNICO, UL-1300A-MSP), followed by the RIE treatment along with a shadow mask to form a hydrophobic bank. After this process, the glass substrate was immersed into a 2-propanol solution containing TDPA (10 mM) for 15 h at 25 °C. The substrate was rinsed with 2-propanol and dried by N_2 gas flow. After the baking process for 30 min at 110 °C, the monolayer-based dielectric layer was formed on the AlOx layer. Source and drain electrodes made of Au (30 nm in thickness) were fabricated by using the thermal evaporation apparatus along with a shadow mask. The polymer semiconductive layer was fabricated by drop-casting a 1,2-dichlorobenzene solution containing PBTTT-C14 (0.0075wt%) onto the channel area (width: 1,000 μm, length: 50 μm). The substrate was annealed at 160 °C for 10 min under the inert atmosphere. Finally, a hydrophobic layer made of CYTOP™ on (CLT-809M in CT-Solv.180, ratio 1:1 (v/v)) for passivation was formed by spin-coating and then baked at 110 °C for 10 min (Figure S1). The reproducibility of the fabricated OFET was evaluated in a previous report.[S1]

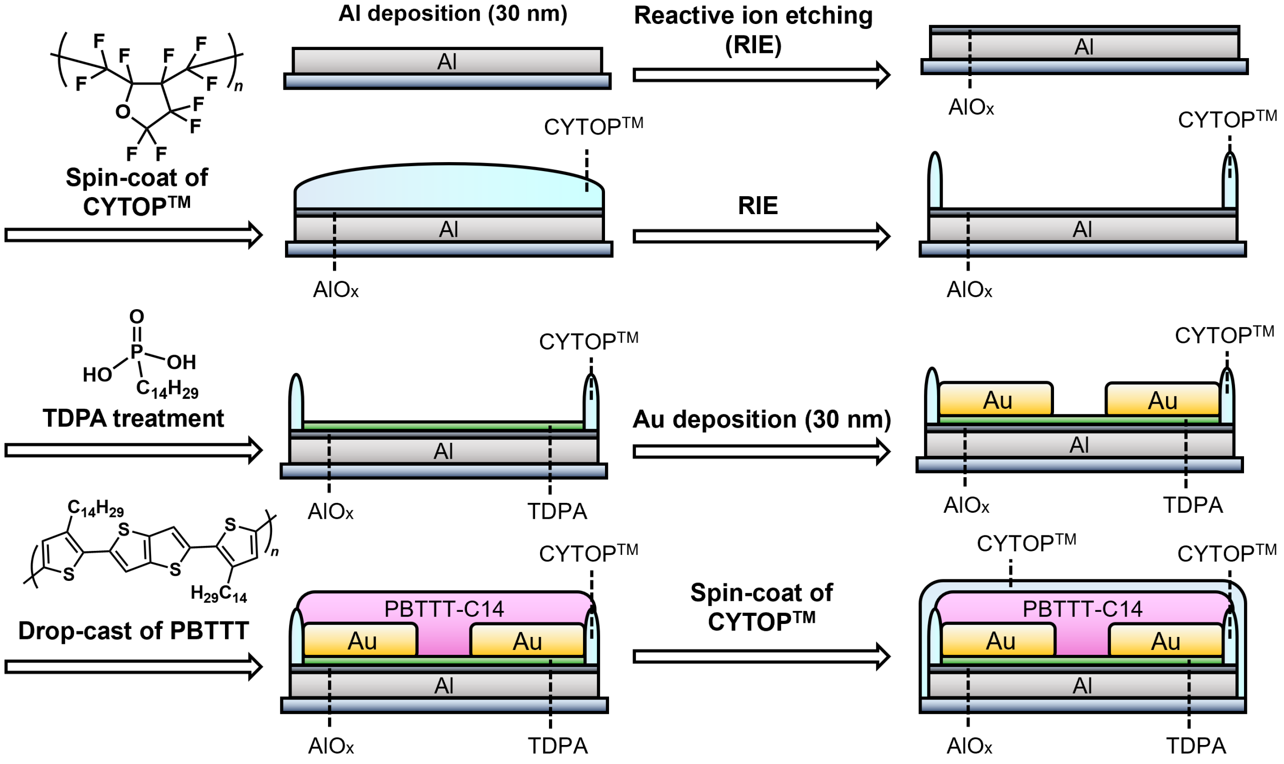

Figure S1. Fabrication scheme for the OFET device.

2.2 Basic OFET characteristics

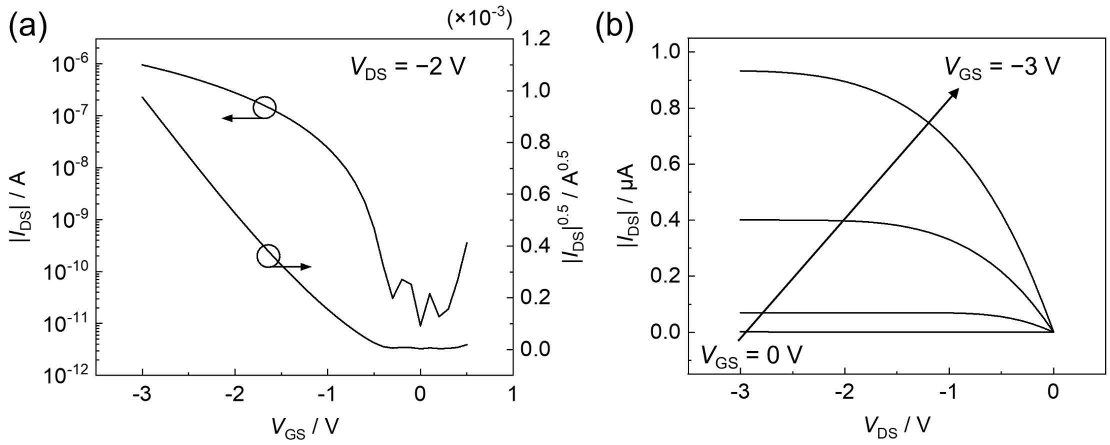

Figure S2. (a) Transfer and (b) output characteristics of the manufactured OFET device.

3. HPLC analysis

The samples for HPLC analysis were treated according to a previously reported method.[S2]

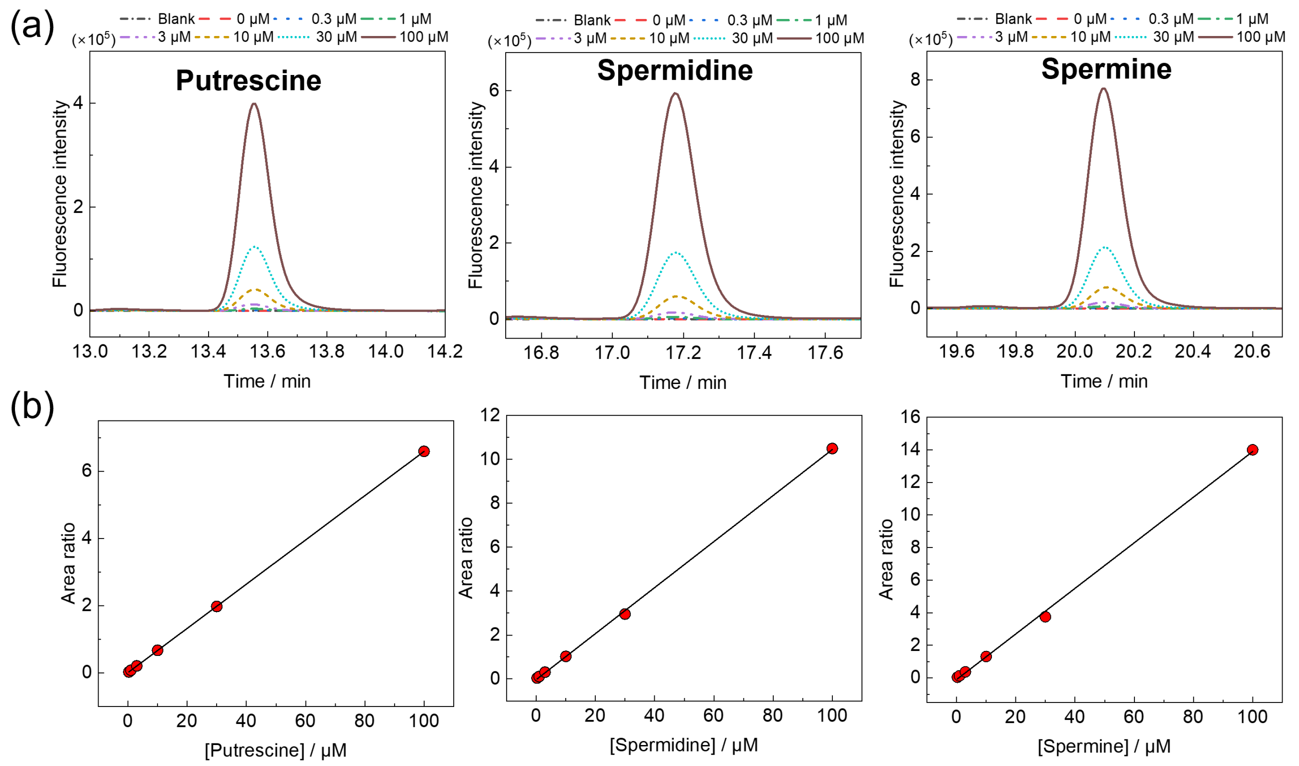

Figure S3. (a) HPLC charts of the standard mixture solution for the establishment of calibration lines. Each peak indicates putrescine, spermidine, and spermine. (b) Correlation between polyamine concentrations and area ratio obtained by HPLC analysis.

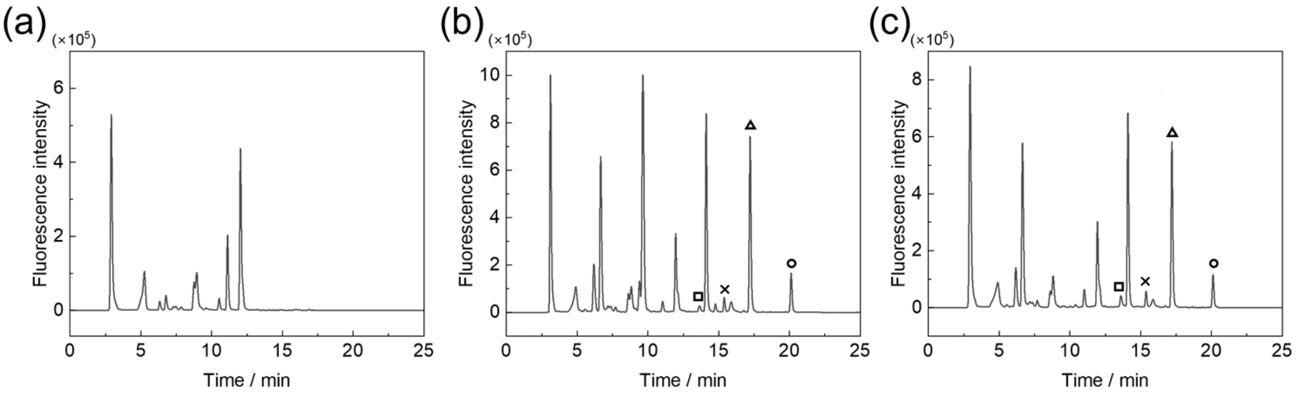

Figure S4. HPLC charts of (a) a control solution (i.e., dansyl chloride after the sample treatment), cosmetic products (b) PHYTOPOLYAMINE^{TM}-S (200 mg/mL), and (c) PHYTOPOLYAMINE^{TM}-SP (10 mg/mL). Each labeled peak indicates 1,7-diaminoheptane (×, internal standard), putrescine (□), spermidine (△), and spermine (○), respectively.

Table S1. Estimated concentrations of polyamines in cosmetic ingredient products by HPLC

Cosmetics	Putrescine (μM)	Spermidine (μM)	Spermine (μM)
PHYTOPOLYAMINE^{TM}-S	5.93	115.78	21.11
PHYTOPOLYAMINE^{TM}-SP	10.55	101.52	16.59

4. Real-sample analysis using the OFET-based chemical sensor

The spike and recovery tests were demonstrated using two cosmetic ingredient products. The PHYTOPOLYAMINE^{TM}-S solutions were mixed with the CHES buffer solution, and their concentrations were 30.7, 47.5, and 67.3 mg/mL. A powder PHYTOPOLYAMINE^{TM}-SP was also dissolved in the CHES buffer, and their concentrations were prepared at 2.8, 3.4, 4.1, and 4.8 mg/mL.

Table S2. Spike and recovery test results for spermidine contained in a cosmetic ingredient product (PHYTOPOLYAMINE^{TM}-SP)

Actual [Spermidine] (μM)	Found [Spermidine] (μM)	Recovery rate (%)
26.52	30.7±0.39	116
33.15	36.4±0.39	110
39.77	41.8±0.78	105
46.40	50.2±1.07	108

Reference

S1. H. Fan, Q. Zhou, R. Mitobe, W. Tang, K. Watanabe, T. Nezaki, N. Nagai and T. Minami, *MRS Commun.* **2022**, *12*, 592–596.

S2. ORYZA POLYAMINE-LC(BG30). https://www.oryza.co.jp/cms/wpcontent/uploads/2019/06/161020-ORYZA-POLYAMINE-LCBG30.pdf (accessed March 22, **2024**).
